# Genetics of Cerebral White Matter Hyperintensities in Diverse Hispanic/Latino Adults

**DOI:** 10.1002/alz70855_104883

**Published:** 2025-12-24

**Authors:** Myriam Fornage, Rui Xia, Tamar Sofer, Carmen R Isasi, Richard B. Lipton, Ariana M Stickel, Wassim Tarraf, Hector M. Gonzalez, Charles Decarli

**Affiliations:** ^1^ University of Texas Health Science Center at Houston, Houston, TX, USA; ^2^ Brigham and Women's Hospital, Boston, MA, USA; ^3^ Harvard Medical School, Boston, MA, USA; ^4^ Albert Einstein College of Medicine, Bronx, NY, USA; ^5^ San Diego State University, San Diego, CA, USA; ^6^ Wayne State University, Detroit, MI, USA; ^7^ University of California San Diego, San Diego, CA, USA; ^8^ University of California Davis, Sacramento, CA, USA

## Abstract

**Background:**

Cerebral white matter hyperintensities (WMH) on magnetic resonance imaging are part of the spectrum of age‐related brain vascular injury and are associated with an increased risk of an increased risk of cognitive impairment and dementia. Genome‐wide association studies (GWAS) conducted mostly in populations of European ancestry have identified several genetic loci. Although Hispanic/Latino adults have a greater burden of WMH than their non‐Hispanics white counterparts, they are vastly underrepresented in genetic studies. We sought to characterize the genetic architecture of WMH in a Hispanic/Latino cohort by investigating the transferability of known WMH genetic loci and by leveraging Hispanic/Latino genetic diversity to map novel loci.

**Methods:**

We conducted genome‐wide association and admixture mapping analyses of WMH volume in a sample of 2159 diverse Hispanic/Latino adults (mean age: 62.4 years; 66% women). We investigated associations at 27 previously‐identified WMH loci. To identify additional loci, we meta‐analyzed our genome‐wide association results with those of the largest GWAS published to date.

**Results:**

WMH heritability was estimated at 29%. Accounting for population differences in linkage disequilibrium, we found some evidence of transferability of 20 of the 27 known WMH loci. Due to power limitations, we could not exclude transferability of the remaining loci. Multi‐ancestry meta‐analysis combining our Hispanic/Latino genome‐wide association results with those from a GWAS of non‐Hispanic white and African American populations identified a novel locus on 12q22 (*p* = 1.8x10^‐8^) tagged by rs10859915, an expression quantitative trait locus of *AMDHD1*. Admixture mapping identified a novel locus on 14q13.2, where higher counts of European ancestry at that locus were significantly associated with higher WMH volume (*p* = 4.9x10^‐7^). This locus spans an 800 kilo‐base region containing *RALGAPA1*, with known impact on neuronal function and brain development. Aggregated rare coding variants in this gene were associated with WMH in a previous analysis of 20,719 stroke and dementia‐free adults.

**Conclusion:**

Our study suggests that WMH loci previously identified in non‐Hispanic white and African American individuals are relevant to Hispanic/Latino adults. It demonstrates the power of the diverse Hispanic/Latino population to fine‐map known genetic loci and discover novel ones, augmenting our understanding of the genetic architecture of cerebral white matter hyperintensities.

